# ﻿Additions to Thelebolales (Leotiomycetes, Ascomycota): *Pseudogeomyceslindneri* gen. et sp. nov. and *Pseudogymnoascuscampensis* sp. nov.

**DOI:** 10.3897/mycokeys.95.97474

**Published:** 2023-02-06

**Authors:** Zhi-Yuan Zhang, Yan-Feng Han, Wan-Hao Chen, Gang Tao

**Affiliations:** 1 College of Eco-Environmental Engineering, Guizhou Minzu University, Guiyang 550025, China Guizhou Minzu University Guiyang China; 2 Institute of Fungus Resources, College of Life Sciences, Guizhou University, Guiyang 550025, China Guizhou University Guiyang China; 3 Basic Medical School, Guizhou University of Traditional Chinese Medicine, Guiyang 550025, China Guizhou University of Traditional Chinese Medicine Guiyang China

**Keywords:** Leotiomycetes, taxonomy, Thelebolales, two new taxa

## Abstract

Thelebolales are globally distributed fungi with diverse ecological characteristics. The classification of Thelebolales remains controversial to date and this study introduces two new taxa, based on morphological and phylogenetic analyses. The results of phylogenetic analyses indicated that the new taxa formed distinct lineages with strong support that were separated from the other members of Thelebolales. The new taxa described herein did not form sexual structures. The phylogenetic relationships of the new taxa and the morphological differences between these taxa and the other species under Thelebolales are also discussed.

## ﻿Introduction

Eriksson and Winka (1997) established the class Leotiomycetes to accommodate the inoperculate discomycetes. Members of this class are ecologically diverse and include saprophytic fungi, endophytic fungi, plant and mammalian pathogens, aquatic and aerial filamentous fungi, mycorrhizal fungi, fungal parasites, root symbionts and wood-rotting fungi, of which the lattermost group mostly includes saprophytic fungi that grow on various substrates (Ekanayaka et al. 2019; Johnston et al. 2019). The order Thelebolales comprises important members of Leotiomycetes due to their diverse functions and potential applications (Hassan et al. 2017; Batista et al. 2020). Thelebolales was established by Haeckel in 1894; however, the classification of this order remains controversial to date (Ekanayaka et al. 2019; Johnston et al. 2019; Batista et al. 2020; Quijada et al. 2022). According to Johnston et al. (2019) and Batista et al. (2020), Thelebolales comprises Pseudeurotiaceae and Thelebolaceae. However, Ekanayaka et al. (2019) reported that Pseudeurotiaceae was nested within Thelebolaceae; thus, the former was regarded as a synonym of the latter. Recently, the work of Quijada et al. (2022) showed that Thelebolaceae is monophyletic and valid, whereas Pseudeurotiaceae is polyphyletic and includes multiple clades and established the Holwayaceae (i.e. *Alatospora*-*Miniancora* clade, Ekanayaka et al. (2019)).

The genus *Pseudogymnoascus* was established by Raillo in 1929; however, a type strain was formally specified during the establishment of the genus. Several years later, Samson (1972) designated *Pseudogymnoascusroseus* Raillo the neotype of *Pseudogymnoascus*, as CBS 395.65 could still be cultivated. At present, the genus *Pseudogymnoascus* comprises 17 valid species (Zhang et al. 2020b; Villanueva et al. 2021; Zhang et al. 2021) belonging to 13 clades (Minnis and Lindner 2013). The genus *Pseudogymnoascus* comprises a diverse group of fungi that are widely distributed on Earth and are highly ecologically diverse.

In this study, two new taxa belonging to the order Thelebolales were isolated in a survey on fungi from urban soil samples in China. This study provides a description, illustrations and a phylogenetic tree for the two new species isolated herein.

## ﻿Materials and methods

### ﻿Fungal isolation and morphology

Soil samples were collected from Cengong County (27°16'98"N, 108°81'46"E) in Kaili City, Guizhou Province, China by Zhi-Yuan Zhang in June 2020. The soil samples were collected from a depth of 3–10 cm from the soil surface. The fungi were isolated using the dilution plate method (Li et al. 2022). Briefly, 2 g of each of the collected samples was suspended in 20 ml of sterile water in a 50 ml sterile conical flask. The conical flasks were thoroughly shaken using a Vortex vibration meter. The suspension was then diluted to a concentration of 10^-4^. Then, 1 ml of the diluted sample was transferred to a sterile Petri dish, following which modified SDA medium (1 g dextrose, 20 g peptone, 20 g agar, and 1 litre ddH_2_O) containing 50 mg/l penicillin and 50 mg/l streptomycin was added and mixed. The experiment was performed in three replicates. The plates were incubated at 25 °C for 1–2 weeks and single colonies were selected from the plates and inoculated on to new potato dextrose agar (**PDA**) plates.

The isolates of potentially new species were transferred to a new plate containing PDA, malt extract agar (**MEA**), oatmeal agar (**OA**) and corn meal agar (**CMA**) and incubated in the dark at 25 °C for 14 days. Photomicrographs of the diagnostic structures were prepared using an OLYMPUS BX53 microscope, equipped with differential interference contrast (**DIC**) optics, an OLYMPUS DP73 high-definition colour camera and cellSens software v.1.18. The dry and living cultures were deposited at the Institute of Fungus Resources, Guizhou University, Guiyang City, Guizhou, China (**GZUIFR**).

### ﻿DNA extraction, PCR amplification and sequencing

The total DNA was extracted using 5% chelex-100 solution. The internal transcribed spacer (**ITS**), nuclear large subunit (**LSU**) rDNA, DNA replication licensing factor (*MCM7*), RNA polymerase II second largest subunit (*RPB2*) and the translation elongation factor EF-1α (*EF1A*) were amplified and sequenced according to the method described by Minnis and Lindner (2013). The sequences of the primers used for amplifying these loci are listed in Suppl. material 1: table S1. The novel sequences identified in this study were deposited in GenBank (Suppl. material 1: table S2).

### ﻿Phylogenetic analyses

The ITS, LSU, *MCM7*, *RPB2* and *EF1A* sequences were retrieved from GenBank, based on previous studies by Zhang et al. (2020b, 2021) and Villanueva et al. (2021) (Suppl. material 1: table S2). The following two datasets were used in this study: (1) the ITS + LSU dataset was used for inferring the phylogenetic placement of the two novel taxa under the order Thelebolales and (2) the ITS + LSU + *MCM7* + *RPB2* + *EF1A* dataset was used for inferring the phylogenetic placement of the new species.

The TBtools software was used for simplifying the nomenclature and renaming (Chen et al. 2020). A single-locus dataset was aligned and edited using MAFFT v.7.037b (Katoh and Standley 2013) and MEGA v.6.06 (Tamura et al. 2013). The “Concatenate Sequence” function in PhyloSuite v1.16 (Zhang et al. 2020a) was used for concatenating each locus. The best-fit substitution model was selected using the corrected Akaike Information Criterion (AICc) in ModelFinder (Kalyaanamoorthy et al. 2017). The combined loci were analysed using the Bayesian Inference (BI) and Maximum Likelihood (ML) methods. The results of ML analysis were implemented in IQ-TREE v.1.6.11 (Nguyen et al. 2015) with 10^4^ bootstrap (BS) tests, using the ultrafast algorithm (Minh et al. 2013). BI analysis was performed with MrBayes v.3.2 (Ronquist et al. 2012) and the Markov Chain Monte Carlo (MCMC) simulations were executed for 10^8^ generations with a sampling frequency every 10^3^ generations and a burn-in of 25%. All the aforementioned analyses were performed in PhyloSuite v.1.16 (Zhang et al. 2020a).

## ﻿Results

### ﻿Phylogenetic analyses

The concatenated alignment of ITS + LSU sequences primarily from the genera under the order Thelebolales comprised 1,209 nucleotides, including inserted gaps (ITS: 433 bp, LSU: 776 bp). The concatenated ITS + LSU + *MCM7* + *RPB2* + *EF1A* dataset from *Pseudogymnoascus* and its related taxa comprised 2,981 nucleotides, including inserted gaps (ITS: 430 bp, LSU: 790 bp, *MCM7*: 475 bp, *RPB2*: 525 bp and *EF1A*: 761 bp). The best-ﬁt evolutionary models obtained by ML and BI analyses of each locus are listed in Suppl. material 1: table S3.

**Figure 1.**
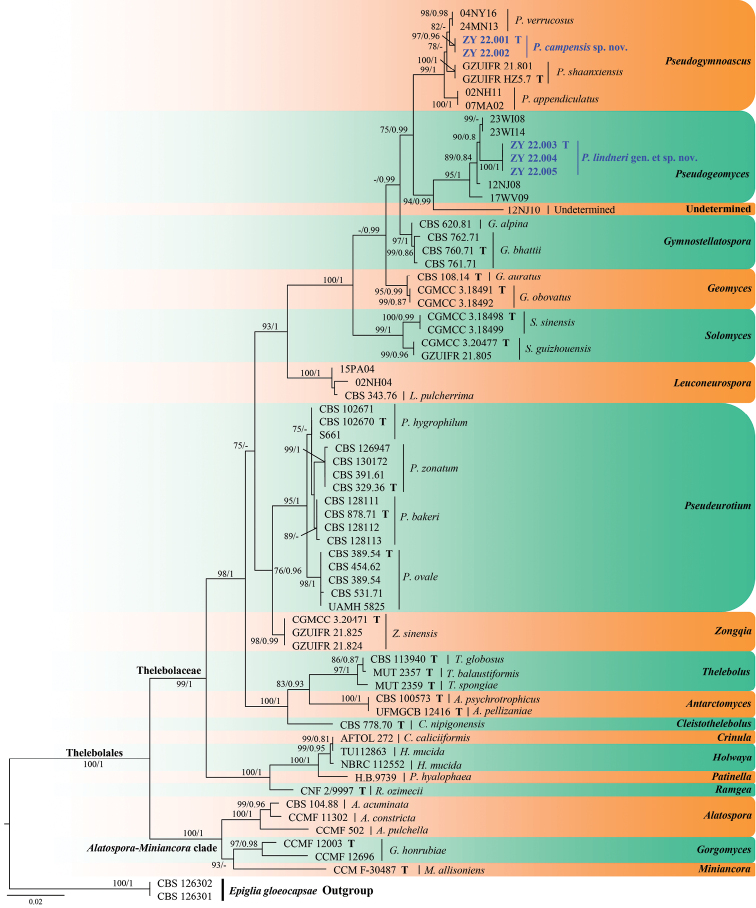
Phylogram generated from a Maximum Likelihood analysis of sequences of Thelebolales, based on ITS and LSU. ML bootstrap values (≥ 75%) and Bayesian posterior probability (≥ 0.75) are indicated along branches (BP/ML). The new taxa are highlighted in bold and blue and “T” indicate ex-type cultures.

The clades formed by the genera in the first phylogenetic tree (Fig. 1) had a high support rate (*Pseudogymnoascus* (100% BS support [BS]/1 posterior probability [PP]), *Solomyces* (100% BS/1 PP), *Pseudogeomyces* (100% BS/1 PP), *Geomyces* (100% BS/1 PP), *Pseudeurotium* (100% BS/1 PP) and *Zongqia* (100% BS/1 PP)). The unidentiﬁed isolate, 12NJ10, formed a single clade (clade N; Minnis and Lindner (2013)) and was separated from the clades formed by the other genera. The new isolates identified in this study were divided into two genera, of which two isolates clustered under the genus *Pseudogymnoascus* and three isolates were clustered under the new genus, *Pseudogeomyces*.

**Figure 2.**
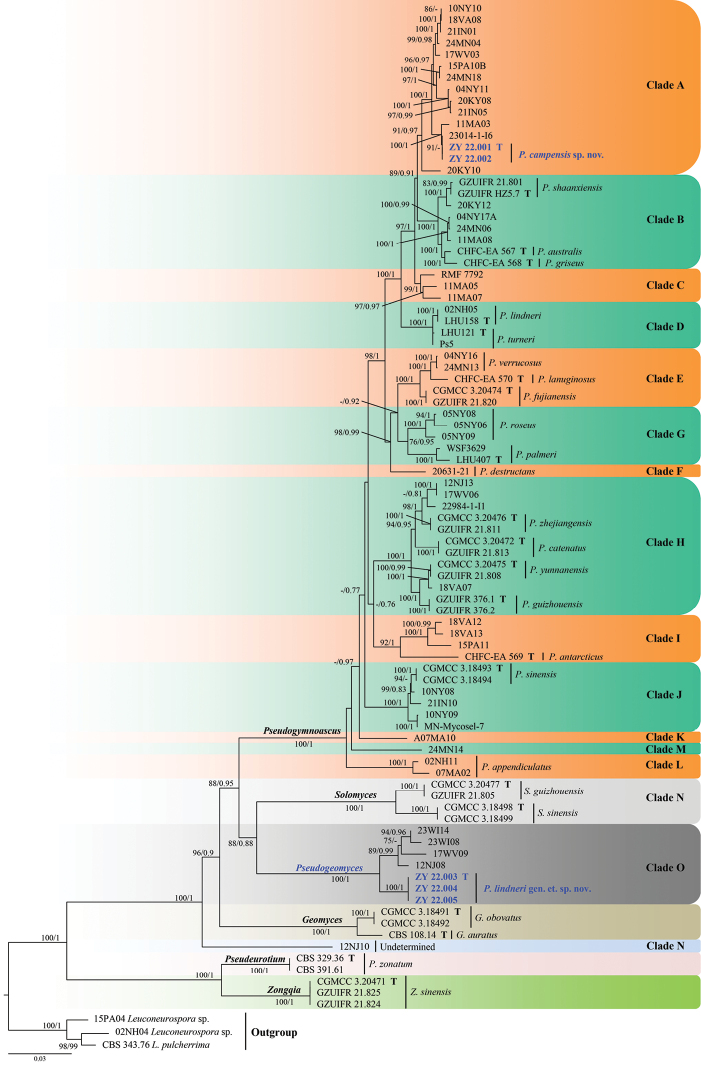
Phylogram generated from A Maximum Likelihood analysis of sequences of Thelebolaceae, based on ITS, LSU, *EF1A*, *RPB2* and *MCM7*. ML bootstrap values (≥ 75%) and Bayesian posterior probability (≥ 0.75) are indicated along branches (BP/ML). Clades are identiﬁed using clade nomenclature (A to O) formally deﬁned by Minnis and Lindner (2013). The new taxa are highlighted in bold and blue and “T” indicate ex-type cultures.

The genera in the second phylogenetic tree (Fig. 2) clustered into monophyletic clades with high support value. The new isolates (ZY 22.003, ZY 22.004 and ZY 22.005) under the new genus, *Pseudogeomyces*, clustered together with the other unidentiﬁed four isolates (12NJ08, 17WV09, 23WI14 and 23WI08) in a well-supported clade (100% BS /1 PP) that was separated from the other clades under Thelebolales. The new isolates, ZY 22.001 and ZY 22.002, belonging to the new species, *Pseudogymnoascuscampensis*, were clustered into a single clade with high support value (97% BS/0.96 PP) under the genus *Pseudogymnoascus*.

### ﻿Taxonomy

#### ﻿ Pseudogeomyces

Taxon classificationFungiThelebolalesThelebolaceae

Zhi.Y. Zhang & Y.F. Han gen. nov.

C875A466-442A-5911-9AB9-2804DDC232FD

 846356

##### Etymology.

Referring to its similarity to *Geomyces*.

##### Geographical distribution.

China and the USA.

##### Description.

***Saprobic*** on the soil. ***Sexual morph***: not observed. ***Asexual morph*: *Hyphae*** branched, septate, smooth. ***Conidiophores*** solitary, rare branches, hyaline, smooth, arising from the erect or geniculated hyphae, usually bearing two to three branches at the tip. ***Conidia*** hyaline, rough, verrucosa, solitary, obovoid, globose to subglobose, borne on hyphae, short protrusions, side branches or in conidiophores separated by connective cells. ***Intercalary conidia*** hyaline, globose to subglobose, fusiform with both truncate. ***Chlamydospores*** not observed.

##### Type species.

*Pseudogeomyceslindneri* Zhi. Y. Zhang & Y. F. Han.

##### Notes.

*Pseudogeomycesis* is introduced to accommodate *Pseudogeomycesislindneri* obtained from urban soil in China and the four isolates (12NJ08, 17WV09, 23WI14 and 23WI08) obtained from bat hibernacular soil in New Jersey, West Virginia and Wisconsin, USA (Minnis and Lindner 2013). Unfortunately, these isolates have not been identified to species to date. Currently, the order Thelebolales consists of 24 genera (Wijayawardene et al. 2017; Ekanayaka et al. 2019; Zhang et al. 2021). The results of phylogenetic analyses (Figs 1, 2) revealed that *Pseudogeomycesis* formed a distinct clade with high support value. However, *Ascophanus*, *Ascozonus*, *Caccobius*, *Coprobolus*, *Leptokalpion*, *Neelakesa* and *Pseudascozonus* are lacking sequence data (Ekanayaka et al. 2019; https://www.ncbi.nlm.nih.gov/, retrieval in Oct 2022); thus, these genera were not included in our phylogenetic analysis. Besides, these genera were reported without asexual morphs (Wijayawardene et al. 2017). Therefore, it was not possible to compare the morphological differences of the newly-established genus, *Pseudogeomycesis* (sexual stage not observed), with the aforementioned genera. However, members of these genera are saprobes (involving dung and wood), terrestrial and widely distributed (Wijayawardene et al. 2017). Of the remaining genera, *Pseudogeomyces* were similar to *Geomyces* and the asexual morphs of *Pseudogymnoascus*. However, *Pseudogeomyces* differed from *Geomyces* and *Pseudogymnoascus* with the presence of two to three irregular branches at the tip of the conidiophores (Kuehn 1958; Van Oorschot 1980).

#### ﻿ Pseudogeomyces lindneri

Taxon classificationFungiThelebolalesThelebolaceae

Zhi. Y. Zhang & Y. F. Han sp. nov.

C915DF68-44FD-52CA-A324-3BC38B91EDB5

 846365

Fig. 3

##### Etymology.

Named after Daniel Lindner, for acknowledging his contributions to the modern taxonomy of *Pseudogymnoascus* and its related taxa.

**Figure 3.**
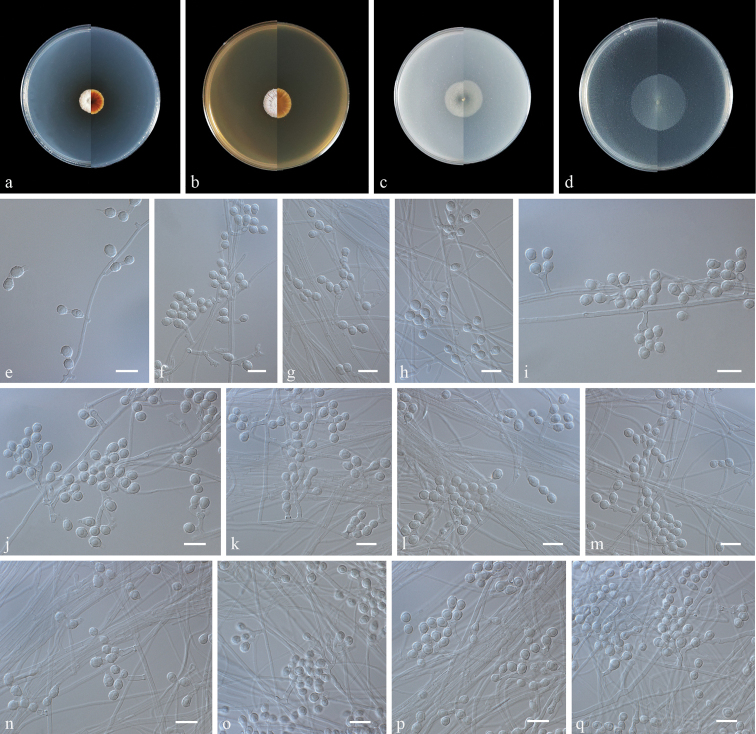
Morphology of *Pseudogeomyceslindneri* sp. nov. **a–d** colony on PDA, MEA, OA and CMA after 14 d at 25 °C (upper surface and lower surface) **e–q** Conidiophore, Conidia and Intercalary conidia. Scale bars: 10 mm (**a–d**); 10 μm (**e–q**).

##### Type.

Kaili City, Guizhou Province, China 27°16'98"N, 108°81'46"E, isolated from the green belt soil, July 2022, Zhi-Yuan Zhang (holotype ZY H-22.003, ex-type ZY 22.003, *ibid.*, ZY 22.004).

##### Geographical distribution.

Guizhou Province, China.

##### Description.

Culture characteristics (14 days at 25 °C): ***Colonies*** on PDA 15–16 mm in diameter, white to pale pink, raised, fluffy, irregular, producing abundant caesious exudates; reverse: brown to cinnamon. ***Colonies*** on MEA 18–19 mm in diameter, off-white, felty, with radial grooves, nearly round, exudates and diﬀusible pigments absent; reverse: brown to cinnamon. ***Colonies*** on OA 25–26 mm in diameter, white, aerial mycelia sparse, flat, nearly round, exudates and diﬀusible pigments absent; reverse: white. ***Colonies*** on CMA 34–35 mm in diameter, white, aerial mycelia sparse, flat, nearly round, margin regular, exudates and diﬀusible pigments absent; reverse: white.

***Hyphae*** hyaline, smooth, branched, septate, 1.0–2.0 μm in diameter. ***Conidiophores*** solitary, rare branches, hyaline, smooth, arising from erect or geniculated hyphae, sometimes reduced to conidiogenous cells, erect, usually bearing two to four conidiogenous cells at the tip. ***Conidia*** hyaline, rough, verrucosa, solitary, obovoid, globose to subglobose, 3.0–7.5 × 2.5–5.5 µm (av. 4.8 × 3.8, n = 50), borne on hyphae, short protrusions, side branches or in conidiophores separated by connective cells. ***Intercalary conidia*** hyaline, globose to subglobose, fusiform, with both truncate 3.5–6.5 × 3.0–4.5 µm (av. 4.9 × 4.0, n = 50). ***Chlamydospores*** not observed. ***Sexual morph*** undetermined.

##### Notes.

Based on multi-locus phylogenetic analyses (Figs 1, 2) and morphological characteristics, *Pseudogeomyceslindneri* is proposed as the type species of *Pseudogeomyces*. The isolates ZY 22.003, ZY 22.004 and ZY 22.005 formed a single phylogenetic clade and were separated from the other four unidentiﬁed isolates (12NJ08, 17WV09, 23WI14 and 23WI08) under *Pseudogeomyces*. Morphologically, *Pseudoge.lindneri* differed from other taxa under the family Thelebolaceae in terms of the presence of two to four irregular branches at the tip of the conidiophores and that the conidia and intercalary conidia are generally connected by connective cells in a chain (Kuehn 1958; Van Oorschot 1980).

#### ﻿ Pseudogymnoascus campensis

Taxon classificationFungiThelebolalesThelebolaceae

Zhi. Y. Zhang & Y. F. Han sp. nov.

EB746C2E-2B9A-5C5D-B7A5-CCD1E0A2AA33

 846366

Fig. 4

##### Etymology.

Refers to Guizhou Minzu University where this fungal type was isolated.

**Figure 4.**
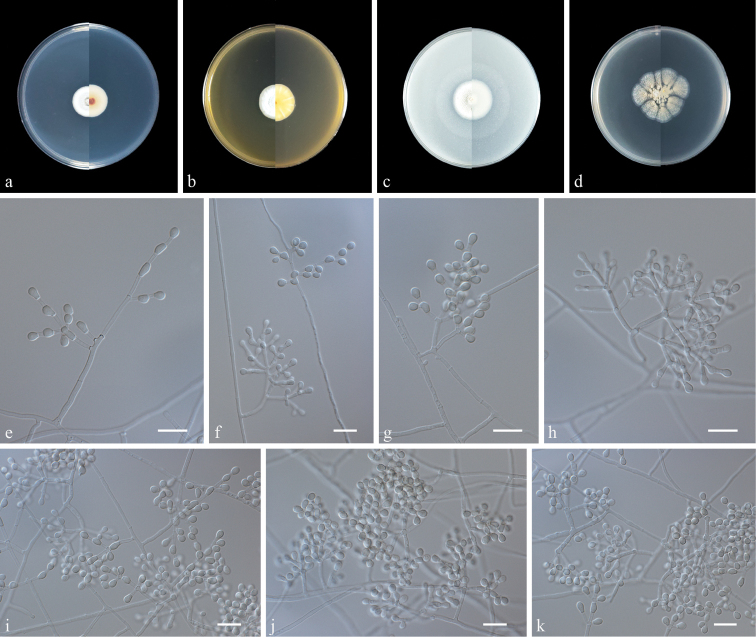
Morphology of *Pseudogymnoascuscampensis* sp. nov. **a–d** colony on PDA, MEA, OA and CMA after 14 d at 25 °C (upper surface and lower surface) **e, f** fertile hyphae bearing arthroconidia and aleurioconidia **g–k** Conidiophore and Conidia. Scale bars: 10 μm (**e–k**).

##### Type.

Guizhou Minzu University, Guiyang City, Guizhou Province, China 26°37'57"N, 106°62'41"E. Colonies form on PDA as a contaminating fungus, July 2022, Zhi-Yuan Zhang (dried holotype ZY H-22.001, ex-type ZY 22.001, *ibid*., ZY 22.002).

##### Geographical distribution.

Guizhou Province, China.

##### Description.

Culture characteristics (14 days at 25 °C): ***Colonies*** on PDA 20–21 mm in diameter, white to light green, fluffy, nearly round, margin regular, exudates and diﬀusible pigments absent; reverse: claret-red to white from centre to margin. ***Colonies*** on MEA 23–24 mm in diameter, white, elevated at the centre, velvety to ﬂoccose, margin regular, exudates and diﬀusible pigments absent; reverse: pale yellow to white. ***Colonies*** on OA 27–28 mm in diameter, white, flat, nearly round, margin regular, exudates absent, producing a diﬀusible faint white pigment; reverse: white. ***Colonies*** on CMA 32–38 mm in diameter, khaki to white, radially sectored by cracks, powdery, exudates and diﬀusible pigments absent; reverse: khaki.

***Hyphae*** hyaline, smooth, branched, septate, 1.0–2.5 μm in diameter. Sometimes lateral hyphae end in barrel-, reniform- or pyriform-shaped chains with blunt-ended arthroconidia, sometimes bearing aleurioconidia, sessile or stalked. ***Conidiophores*** abundant, solitary, erect, arising in acute angles with the main axis, hyaline, smooth, usually bearing verticils of two to three branches arising from the stipe at an acute angle. ***Aleurioconidia*** pyriform or obovoid, with a broad truncated basal scar, 3.0–5.0 × 2.0–2.5 µm (av. 3.6 × 2.7, n = 50), in conidiophores separated by connective cells, smooth or rough. ***Intercalary conidia*** barrel, reniform, pyriform to elongated or irregular, with a broad truncated scar at the base or both ends, 3.5–5.5 × 2.0–3.0 µm (av. 4.0 × 2.6, n = 50), smooth or rough. ***Arthroconidia*** not observed. ***Sexual morph*** unknown.

##### Notes.

Minnis and Lindner (2013) proposed multiple clades of *Pseudogymnoascus* and allies (clades A to O), based on phylogenetic analyses using North American isolates. In this study, *Pseudogymnoascuscampensis* was placed in clade A (Fig. 1). Clade A harbours 13 isolates for which no morphological data are yet available and remain as unidentiﬁed species to date (Minnis and Lindner 2013; Leushkin et al. 2015). These isolates were obtained from bat hibernacular soil in the USA (Minnis and Lindner 2013). *Pseudogymnoascuscampensis* (ZY 22.001 and ZY 22.002), 23014-1-I6 and 11MA03 formed an independent lineage with strong support (MLBS 100/PP 1, Fig. 1). The closest known species to *Pseudogy.campensis* are *Pseudogy.shaanxiensis*, *Pseudogy.australis* and *Pseudogy.griseus*, which are members of the neighbouring clade B (Zhang et al. 2020b, Villanueva et al. 2021). However, *Pseudogy.campensis* can be distinguished from *Pseudogy.shaanxiensis*, *Pseudogy.australis* and *Pseudogy.griseus* by the absence of exudates on PDA, MEA and CMA media and lack of arthroconidia (Zhang et al. 2020b; Villanueva et al. 2021).

## ﻿Discussion

Previously, Minnis and Lindner (2013) performed a phylogenetic analysis, based on numerous multi-loci sequences of *Pseudogymnoascus* and its allies isolated from North American cave soils and obtained very robust results. However, many of the isolates obtained in the study were not identified as species. Based on their work, we subsequently defined *Pseudogymnoascus* and its allies isolated from China and reported two new genera and several new species (Zhang et al. 2020b, 2021). Similarly, Villanueva et al. (2021) identified four strains isolated from Antarctica, based on the above study and found that they were all previously undescribed species. In this study, one new genus and one new species are being proposed, based on the aforementioned study.

The classification of Thelebolales remains controversial to date (Ekanayaka et al. 2019; Johnston et al. 2019; Batista et al. 2020; Quijada et al. 2022). In contrast, however, the work of Ekanayaka et al. (2019) contained more genera in Thelebolales; therefore, we continued the phylogenetic analysis in Thelebolales, based on this study. This study, based on ITS+LSU phylogenetic analyses, showed that Thelebolales consisted of Thelebolaceae and *Alatospora*-*Miniancora* clade (Fig. 1), which is consistent with Ekanayaka et al. (2019). Our proposed new genus *Pseudogeomyces* was nested in Thelebolaceae and is well supported (Fig. 1).

The ITS region is the most frequently used molecular marker in fungal classification studies, primarily due to its suitable variability. Additionally, Vu et al. (2019) demonstrated the high efficacy of ITS and LSU concatenation in discriminating ﬁlamentous fungal species. Numerous fungal ITS and LSU sequences are presently available in public databases (Zhang et al. 2022). Additionally, some fungal taxa, including the majority of genera under Thelebolales, have only ITS and/or LSU regions. Therefore, we only explored the position of the new genus, *Pseudogeomyces*, in Thelebolales, based on the phylogenetic analysis of ITS + LSU sequences.

In accordance with the most recent revision to the rules governing fungal nomenclature, presently referred to as the “International Code of Nomenclature for algae, fungi and plants”, the system of dual nomenclature sanctioned by Article 59 has been modified to ‘‘One Fungus, One Name’’ (McNeill et al. 2012), where a single name is applied, regardless of the life stage considered. Most of the new taxa erected in recent years under *Pseudogymnoascus* and allies are based on asexual structures rather than sexual structures (Zhang et al. 2020b; Villanueva et al. 2021; Zhang et al. 2021). In this study, the new isolates were separately cultured in four media for observing the sexual structures, but the approach proved unsuccessful. The sexual structures of fungi appear when grown in nature rather than under laboratory conditions. Therefore, studying the production of sexual structures by these fungi under laboratory conditions is highly necessary.

## Supplementary Material

XML Treatment for Pseudogeomyces

XML Treatment for Pseudogeomyces lindneri

XML Treatment for Pseudogymnoascus campensis

## Supplementary materials

Supplementary material 1 Sequences of primers used for the amplification of molecular markers in this study. GenBank accession numbers of the sequences used in this study. The best-fit evolutionary model in the phylogenetic analyses Data type table (word file) https://binary.pensoft.net/file/806106This dataset is made available under the Open Database License (http://opendatacommons.org/licenses/odbl/1.0/). The Open Database License (ODbL) is a license agreement intended to allow users to freely share, modify, and use this Dataset while maintaining this same freedom for others, provided that the original source and author(s) are credited.Zhi-Yuan Zhang, Yan-Feng Han, Wan-Hao Chen, Gang Tao
