# Supplementary material for: ﻿Additions to Thelebolales (Leotiomycetes, Ascomycota): Pseudogeomyceslindneri gen. et sp. nov. and Pseudogymnoascuscampensis sp. nov
Source: MycoKeys. 2023 Feb 6;95:47–60. doi: 10.3897/mycokeys.95.97474 (PMC10210254; doi:10.3897/mycokeys.95.97474)
Supplement: Supplementary material 1 — Sequences of primers used for the amplification of molecular markers in this study. GenBank accession numbers of the sequences used in this study. The best-fit evolutionary model in the phylogenetic analyses [file mycokeys-95-047-s001.docx]

**Table S1.** Sequences of primers used for the amplification of molecular markers in this study.

| Molecular marker | Primer name | Primer sequence (5´-3´) | Reference |
| --- | --- | --- | --- |
| ITS | ITS1 | TCCGTAGGTGAACCTGCG | White et al. 1990 |
|  | ITS4 | TCCTCCGCTTATTGATATGC | White et al. 1990 |
| LSU | LR0R | ACCCGCTGAACTTAAGC | Moncalvo et al. 2000 |
|  | LR7 | TACTACCACCAAGATCT | Vilgalys and Hester 1990 |
| *EF1A* | 2218R | ATGACACCRACRGCRACRGTYTG | Rehner and Buckley 2005 |
|  | 983F | GCYCCYGGHCAYCGTGAYTTYAT | Rehner and Buckley 2005 |
| *MCM7* | Mcm7-709for | ACNMGNGTNTCVGAYGTHAARCC | Schmitt et al. 2009 |
|  | Mcm7-1348rev | GAYTTDGCNACNCCNGGRTCWCCCAT | Schmitt et al. 2009 |
| *RPB2* | fRPB2-7cF | ATGGGYAARCAAGCYATGGG | Liu et al. 1999 |
|  | RPB2-3053bR | TGRATYTTRTCRTCSACCAT | Reeb et al. 2004 |

ITS: internal transcribed spacer; LSU: nuclear large subunit rDNA; *MCM7*: DNA replication licensing; *RPB2*: RNA polymerase II second largest subunit; *EF1A*: translation elongation factor EF-1a.

References

White TJ, Bruns T, Lee S, Taylor J (1990) Amplification and direct sequencing of fungal ribosomal RNA genes for phylogenetics. In: Innis MA, Gelfand DH, Sninsky JJ, White TJ (Eds.) PCR protocols: a guide to methods and applications, Academic Press, San Diego, California, 315–322. <https://doi.org/10.1016/b978-0-12-372180-8.50042-1>

Moncalvo JM, Lutzoni FM, Rehner SA, Johnson J, Vilgalys R (2000). Phylogenetic relationships of agaric fungi based on nuclear large subunit ribosomal DNA sequences. Systematic Biology 49: 278–305. <https://doi.org/10.1080/10635159950173852>

Vilgalys R, Hester M (1990) Rapid genetic identification and mapping of enzymatically amplified ribosomal DNA from several Cryptococcus species. Journal of Bacteriology 172: 4238–4246. <https://doi.org/10.1128/jb.172.8.4238-4246.1990>

Rehner SA, Buckley E (2005) A *Beauveria* phylogeny inferred from nuclear ITS and EF1-α sequences: Evidence for cryptic diversification and links to *Cordyceps* teleomorphs. Mycologia 97: 84–98. <https://doi.org/10.1080/15572536.2006.11832842>

Schmitt I, Crespo A, Divakar PK, Fankhauser JD, Herman-Sackett E, Kalb K. et al. (2009). New primers for promising single-copy genes in fungal phylogenetics and systematics. Persoonia 23: 35–40. <https://doi.org/10.3767/003158509x470602>

Liu YJ, Whelen S, Hall BD (1999) Phylogenetic relationships among Ascomycetes: Evidence from an RNA polymerse II subunit. Molecular Biology and Evolution 16: 1799–1808. <https://doi.org/10.1093/oxfordjournals.molbev.a026092>

Reeb V, Lutzoni F, Roux C (2004). Contribution of RPB2 to multilocus phylogenetic studies of the euascomycetes (Pezizomycotina, Fungi) with special emphasis on the lichen-forming Acarosporaceae and evolution of polyspory. Molecular Phylogenetics and Evolution 32: 1036–1060. <https://doi.org/10.1016/j.ympev.2004.04.012>

**Table S2.** GenBank accession numbers of the sequences used in this study.

| Species | Strains | ITS | LSU | *MCM7* | *RPB2* | *EF1A* | Used in Trees |
| --- | --- | --- | --- | --- | --- | --- | --- |
| *Alatospora acuminata* | CBS 104.88 | MH862121 | MH873811 |  |  |  | 1 |
| *Alatospora constricta* | CCM F-11302 | KC834040 | KC834017 |  |  |  | 1 |
| *Alatospora pulchella* | CCM F-502 | KC834039 | KC834019 |  |  |  | 1 |
| *Antarctomyces pellizaniae* | UFMGCB 12416 **T** | KX576510 |  |  |  |  | 1 |
| *Antarctomyces psychrotrophicus* | CBS 100573 **T** | MH874317 |  |  |  |  | 1 |
| *Cleistothelebolus nipigonensis* | CBS 778.70 **T** | MH859938 | MH871738 |  |  |  | 1 |
| *Crinula caliciiformis* | AFTOL-ID 272 | KT225524 | AY544680 |  |  |  | 1 |
| *Epiglia gloeocapsae* | CBS 126301 | MH863968 | MH875423 |  |  |  | 1 |
| *Epiglia gloeocapsae* | CBS 126302 | MH863969 | MH875424 |  |  |  | 1 |
| *Geomyces auratus* | CBS 108.14 **T** | KF039895 | KF017864 | KF017690 | KF017746 | KF017805 | 1/2 |
| *Geomyces obovatus* | CGMCC 3.18491 **T** | MT509362 | MT509376 | MT534202 | MT534216 | MT534227 | 1/2 |
| *Geomyces obovatus* | CGMCC 3.18492 | MT509363 | MT509377 | MT534203 | MT534217 | MT534228 | 1/2 |
| *Gorgomyces honrubiae* | CCM F-12003 **T** | KC834057 | KC834028 |  |  |  | 1 |
| *Gorgomyces honrubiae* | CCM F-12696 | KC834058 |  |  |  |  | 1 |
| *Gymnostellatospora alpina* | CBS 620.81 | MH861383 | MH873132 |  |  |  | 1 |
| *Gymnostellatospora bhattii* | CBS 760.71 **T** | MH860337 | MH872092 |  |  |  | 1 |
| *Gymnostellatospora bhattii* | CBS 761.71 | MH860338 | MH872093 |  |  |  | 1 |
| *Gymnostellatospora bhattii* | CBS 762.71 | MH860339 | MH872094 |  |  |  | 1 |
| *Holwaya mucida* | NBRC 112552 | LC425042 | LC429385 |  |  |  | 1 |
| *Holwaya mucida* | TU 112863 | MH752062 | KX090844 |  |  |  | 1 |
| *Leuconeurospora pulcherrima* | CBS 343.76 | KF049206 | FJ176884 |  | FJ238367 | FJ238409 | 1/2 |
| *Leuconeurospora* sp. | 02NH04 | JX270349 | KF017817 | KF017648 | KF017702 | KF017757 | 1/2 |
| *Leuconeurospora* sp. | 15PA04 | JX270479 | KF017841 | KF017669 | KF017725 | KF017781 | 1/2 |
| *Miniancora allisoniens* | CCM F-30487 **T** | KC834064 |  |  |  |  | 1 |
| *Patinella hyalophaea* | H.B.9739 | KT876978 | KT876978 |  |  |  | 1 |
| *Pseudeurotium bakeri* | CBS 878.71 **T** | MH860393 | MH872136 |  |  |  | 1 |
| *Pseudeurotium bakeri* | CBS 128111 | MH864831 | MH876274 |  |  |  | 1 |
| *Pseudeurotium bakeri* | CBS 128112 | MH864832 | MH876275 |  |  |  | 1 |
| *Pseudeurotium bakeri* | CBS 128113 | MH864833 | MH876276 |  |  |  | 1 |
| *Pseudeurotium hygrophilum* | CBS 102670 **T** | AY129291 | MH874401 |  |  |  | 1 |
| *Pseudeurotium hygrophilum* | CBS 102671 | AY129292 |  |  |  |  | 1 |
| *Pseudeurotium hygrophilum* | S661 | KP644137 | KP644138 |  |  |  | 1 |
| *Pseudeurotium ovale* | CBS 389.54 **T** | MH857368 | MH868913 |  |  |  | 1 |
| *Pseudeurotium ovale* | CBS 454.62 | MH858209 | MH869809 |  |  |  | 1 |
| *Pseudeurotium ovale* | CBS 531.71 | MH860256 | MH872019 |  |  |  | 1 |
| *Pseudeurotium ovale* | CBS 389.54 | AY129289 |  |  |  |  | 1 |
| *Pseudeurotium ovale* | UAMH 5825 | KJ755521 |  |  |  |  | 1 |
| *Pseudeurotium zonatum* | CBS 329.36 **T** | AY129286 | DQ470988 |  | DQ470940 | DQ471112 | 1/2 |
| *Pseudeurotium zonatum* | CBS 391.61 | MH858096 | MH869666 |  |  |  | 1/2 |
| *Pseudeurotium zonatum* | CBS 126947 | MH864346 | MH875790 |  |  |  | 1 |
| *Pseudeurotium zonatum* | CBS 130172 | MH865520 | MH876956 |  |  |  | 1 |
| ***Pseudogeomyces lindneri*** | **ZY 22.003 T** | **OP796797** | **OP796792** | **OP781436** |  | **OP781431** | 1/2 |
| ***Pseudogeomyces lindneri*** | **ZY 22.004** | **OP796798** | **OP796793** | **OP781437** |  | **OP781432** | 1/2 |
| ***Pseudogeomyces lindneri*** | **ZY 22.005** | **OP796799** | **OP796794** | **OP781438** |  | **OP781433** | 1/2 |
| *Pseudogeomyces* sp. | 12NJ08 | JX270454 | KF017836 | KF017665 |  | KF017776 | 1/2 |
| *Pseudogeomyces* sp. | 17WV09 | JX270515 | KF017846 | KF017674 |  | KF017787 | 1/2 |
| *Pseudogeomyces* sp. | 23WI08 | JX270598 | KF017858 |  |  | KF017799 | 1/2 |
| *Pseudogeomyces* sp. | 23WI14 | JX270604 |  | KF017684 |  |  | 1/2 |
| *Pseudogymnoascus antarcticus* | CHFC-EA 569 **T** | JX845280 | MN417282 | MN432493 | MN418135 | MN418131 | 2 |
| *Pseudogymnoascus appendiculatus* | 02NH11 | JX270356 | KF017819 | KF017650 | KF017704 | KF017759 | 1/2 |
| *Pseudogymnoascus appendiculatus* | 07MA02 | JX270402 | KF017827 | KF017658 | KF017712 | KF017767 | 1/2 |
| *Pseudogymnoascus australis* | CHFC-EA 567 **T** | MN417287 | MN417284 | MN432491 | MN418137 | MN418133 | 1 |
| ***Pseudogymnoascus campensis*** | **ZY 22.001 T** | **OP796795** | **OP796790** | **OP781434** |  | **OP781429** | 1/2 |
| ***Pseudogymnoascus campensis*** | **ZY 22.002** | **OP796796** | **OP796791** | **OP781435** |  | **OP781430** | 1/2 |
| *Pseudogymnoascus catenatus* | GZUIFR 21.813 | MZ444078 | MZ444105 | MZ490760 | MZ488543 | MZ488520 | 2 |
| *Pseudogymnoascus catenatus* | CGMCC 3.20472 **T** | MZ444080 | MZ444107 | MZ490762 | MZ488545 | MZ488522 | 2 |
| *Pseudogymnoascus destructans* | 20631-21 | EU884921 | KF017865 | KF017691 | KF017747 | KF017806 | 2 |
| *Pseudogymnoascus fujianensis* | CGMCC 3.20474 **T** | MZ444084 | MZ444111 | MZ490766 | MZ488549 | MZ488526 | 2 |
| *Pseudogymnoascus fujianensis* | GZUIFR 21.820 | MZ444085 | MZ444112 | MZ490767 | MZ488550 | MZ488527 | 2 |
| *Pseudogymnoascus griseus* | CHFC-EA 568 **T** | MN417288 | MN417285 | MN432492 | MN418138 | MN418134 | 2 |
| *Pseudogymnoascus guizhouensis* | GZUIFR 376.1 **T** | MT509369 | MT509383 | MT534209 | MT534223 | MT534234 | 2 |
| *Pseudogymnoascus guizhouensis* | GZUIFR 376.2 | MT509370 | MT509384 | MT534210 | MT534224 | MT534235 | 2 |
| *Pseudogymnoascus lanuginosus* | CHFC-EA 570 **T** | MN417286 | MN417283 | MN418139 | MN418136 | MN418132 | 2 |
| *Pseudogymnoascus lindneri* | 02NH05 | JX270350 | KF017818 | KF017649 | KF017703 | KF017758 | 2 |
| *Pseudogymnoascus lindneri* | LHU158 **T** | MN542212 |  |  | MN541384 | MN541383 | 2 |
| *Pseudogymnoascus palmeri* | WSF 3629 | KF039897 | KF017870 | KF017696 | KF017751 | KF017811 | 2 |
| *Pseudogymnoascus palmeri* | LHU407 **T** | MT988150 |  |  | MW054468 | MW054467 | 2 |
| *Pseudogymnoascus roseus* | 05NY06 | JX270385 | KF017824 | KF017655 | KF017709 | KF017764 | 2 |
| *Pseudogymnoascus roseus* | 05NY08 | JX270387 | KF017825 | KF017656 | KF017710 | KF017765 | 2 |
| *Pseudogymnoascus roseus* | 05NY09 | JX270388 | KF017826 | KF017657 | KF017711 | KF017766 | 2 |
| *Pseudogymnoascus shaanxiensis* | GZUIFR HZ5.7 **T** | MT509366 | MT509380 | MT534206 | MT534220 | MT534231 | 1/2 |
| *Pseudogymnoascus shaanxiensis* | GZUIFR 21.801 | MZ444066 | MZ444093 | MZ490748 | MZ488531 | MZ488508 | 1/2 |
| *Pseudogymnoascus sinensis* | CGMCC 3.18493 **T** | MT509364 | MT509378 | MT534204 | MT534218 | MT534229 | 2 |
| *Pseudogymnoascus sinensis* | CGMCC 3.18494 | MT509365 | MT509379 | MT534205 | MT534219 | MT534230 | 2 |
| *Pseudogymnoascus* sp. | 04NY11 | JX270375 | KF017821 | KF017652 | KF017706 | KF017761 | 2 |
| *Pseudogymnoascus* sp. | 04NY17A | JX270378 | KF017823 | KF017654 | KF017708 | KF017763 | 2 |
| *Pseudogymnoascus* sp. | 10NY08 | JX270432 | KF017829 | KF017659 | KF017714 | KF017769 | 2 |
| *Pseudogymnoascus* sp. | 10NY09 | JX270433 | KF017830 | KF017660 | KF017715 | KF017770 | 2 |
| *Pseudogymnoascus* sp. | 10NY10 | JX270434 | KF017831 |  | KF017716 | KF017771 | 2 |
| *Pseudogymnoascus* sp. | 11MA03 | JX270438 | KF017832 | KF017661 | KF017717 | KF017772 | 2 |
| *Pseudogymnoascus* sp. | 11MA05 | JX270440 | KF017833 | KF017662 | KF017718 | KF017773 | 2 |
| *Pseudogymnoascus* sp. | 11MA07 | JX270442 | KF017834 | KF017663 | KF017719 | KF017774 | 2 |
| *Pseudogymnoascus* sp. | 11MA08 | JX270443 | KF017835 | KF017664 | KF017720 | KF017775 | 2 |
| *Pseudogymnoascus* sp. | 12NJ13 | JX270459 | KF017838 | KF017667 | KF017722 | KF017778 | 2 |
| *Pseudogymnoascus* sp. | 15PA11 | JX270486 | KF017843 | KF017671 | KF017727 | KF017783 | 2 |
| *Pseudogymnoascus* sp. | 17WV03 | JX270510 | KF017844 | KF017672 | KF017728 | KF017784 | 2 |
| *Pseudogymnoascus* sp. | 17WV06 | JX270513 |  | KF017673 | KF017729 | KF017785 | 2 |
| *Pseudogymnoascus* sp. | 18VA07 | JX270527 | KF017847 | KF017675 |  | KF017788 | 2 |
| *Pseudogymnoascus* sp. | 18VA08 | JX270528 | KF017848 | KF017676 | KF017731 | KF017789 | 2 |
| *Pseudogymnoascus* sp. | 18VA12 | JX270532 | KF017849 |  | KF017732 | KF017790 | 2 |
| *Pseudogymnoascus* sp. | 18VA13 | JX270533 | KF017850 |  | KF017733 | KF017791 | 2 |
| *Pseudogymnoascus* sp. | 20KY08 | JX270562 | KF017851 | KF017677 | KF017734 | KF017792 | 2 |
| *Pseudogymnoascus* sp. | 20KY10 | JX270563 | KF017852 | KF017678 | KF017735 | KF017793 | 2 |
| *Pseudogymnoascus* sp. | 20KY12 | JX270565 | KF017853 | KF017679 | KF017736 | KF017794 | 2 |
| *Pseudogymnoascus* sp. | 21IN01 | JX270568 | KF017854 | KF017680 | KF017737 | KF017795 | 2 |
| *Pseudogymnoascus* sp. | 21IN05 | JX270572 | KF017855 | KF017681 | KF017738 | KF017796 | 2 |
| *Pseudogymnoascus* sp. | 21IN10 | JX270577 | KF017856 | KF017682 | KF017739 | KF017797 | 2 |
| *Pseudogymnoascus* sp. | 24MN04 | JX270612 | KF017859 | KF017685 | KF017741 | KF017800 | 2 |
| *Pseudogymnoascus* sp. | 24MN06 | JX270614 | KF017860 | KF017686 | KF017742 | KF017801 | 2 |
| *Pseudogymnoascus* sp. | 24MN14 | JX270622 | KF017862 | KF017688 | KF017744 | KF017803 | 2 |
| *Pseudogymnoascus* sp. | 24MN18 | JX270626 | KF017863 | KF017689 | KF017745 | KF017804 | 2 |
| *Pseudogymnoascus* sp. | 22984-1-I1 | JX415262 | KF017866 | KF017692 |  | KF017807 | 2 |
| *Pseudogymnoascus* sp. | 23014-1-I6 | JX512256 | KF017867 | KF017693 | KF017748 | KF017808 | 2 |
| *Pseudogymnoascus* sp. | A07MA10 | KF039893 | KF017828 |  | KF017713 | KF017768 | 2 |
| *Pseudogymnoascus* sp. | 15PA10B | KF039894 | KF017842 | KF017670 | KF017726 | KF017782 | 2 |
| *Pseudogymnoascus* sp. | RMF 7792 | KF039898 | KF017871 | KF017697 | KF017752 | KF017812 | 2 |
| *Pseudogymnoascus* sp. | MN-Mycosel-7 | KF039899 | KF017872 | KF017698 | KF017753 | KF017813 | 2 |
| *Pseudogymnoascus turneri* | LHU 121 **T** | MN542213 |  |  | MN541380 | MN541379 | 2 |
| *Pseudogymnoascus turneri* | Ps5 | MN542214 |  |  | MN541382 | MN541381 | 2 |
| *Pseudogymnoascus verrucosus* | 04NY16 | JX270377 | KF017822 | KF017653 | KF017707 | KF017762 | 1/2 |
| *Pseudogymnoascus verrucosus* | 24MN13 | JX270621 | KF017861 | KF017687 | KF017743 | KF017802 | 1/2 |
| *Pseudogymnoascus yunnanensis* | CGMCC 3.20475 **T** | MZ444072 | MZ444099 | MZ490754 | MZ488537 | MZ488514 | 2 |
| *Pseudogymnoascus yunnanensis* | GZUIFR 21.808 | MZ444073 | MZ444100 | MZ490755 | MZ488538 | MZ488515 | 2 |
| *Pseudogymnoascus zhejiangensis* | CGMCC 3.20476 **T** | MZ444075 | MZ444102 | MZ490757 | MZ488540 | MZ488517 | 2 |
| *Pseudogymnoascus zhejiangensis* | GZUIFR 21.811 | MZ444076 | MZ444103 | MZ490758 | MZ488541 | MZ488518 | 2 |
| *Ramgea ozimecii* | CNF 2/9997 **T** | KY368752 | KY368753 |  |  |  | 1 |
| *Solomyces guizhouensis* | CGMCC 3.20477 **T** | MZ444069 | MZ444096 | MZ490751 | MZ488534 | MZ488511 | 1/2 |
| *Solomyces guizhouensis* | GZUIFR 21.805 | MZ444070 | MZ444097 | MZ490752 | MZ488535 | MZ488512 | 1/2 |
| *Solomyces sinensis* | CGMCC 3.18498 **T** | MT509373 | MT509387 | MT534213 |  | MT534238 | 1/2 |
| *Solomyces sinensis* | CGMCC 3.18499 | MT509374 | MT509388 | MT534214 |  | MT534239 | 1/2 |
| *Thelebolus balaustiformis* | MUT 2357 **T** | NR_159056 | NG_067559 |  |  |  | 1 |
| *Thelebolus globosus* | CBS 113940 **T** | MH862951 | NG_067263 |  |  |  | 1 |
| *Thelebolus spongiae* | MUT 2359 **T** | MG813185 | MG816493 |  |  |  | 1 |
| Undetermined | 12NJ10 | JX270456 | KF017837 | KF017666 | KF017721 | KF017777 | 1/2 |
| *Zongqia sinensis* | CGMCC 3.20471 **T** | MZ444088 | MZ444115 | MZ490770 | MZ488553 |  | 1/2 |
| *Zongqia sinensis* | GZUIFR 21.824 | MZ444089 | MZ444116 | MZ490771 | MZ488554 |  | 1/2 |
| *Zongqia sinensis* | GZUIFR 21.825 | MZ444090 | MZ444117 | MZ490772 | MZ488555 |  | 1/2 |

Ex-type strains are indicated with **T**. Sequences highlighted in **bold and blue** were generated in this study.

**Table S3.** The best-fit evolutionary model in the phylogenetic analyses.

| **Dataset** | **Phylogenetic analysis** | **Model** | | | | |
| --- | --- | --- | --- | --- | --- | --- |
|  |  | **ITS** | **LSU** | ***MCM7*** | ***RPB2*** | ***EF1A*** |
| First dataset | ML analysis | SYM+I+G4 | TIM+F+I+G4 |  |  |  |
|  | BI analysis | SYM+I+G4 | GTR+F+I+G4 |  |  |  |
| Second dataset | ML analysis | TIM2e+I+G4 | GTR+F+I | TVMe+I+G4 | TIM3e+I+G4 | GTR+F+I+G4 |
|  | BI analysis | SYM+I+G4 | GTR+F+I | SYM+I+G4 | K2P+G4 | GTR+F+I+G4 |

ML: Maximum likelihood; BI: Bayesian inference.
